# Racial and ethnic disparities in diabetes-related healthcare service use

**DOI:** 10.3389/fcdhc.2026.1675970

**Published:** 2026-05-18

**Authors:** Gaole Song, C. Anderson Johnson, Wei-Chin Hwang, Yawen Li, Bin Xie

**Affiliations:** 1 Department of Population Sciences, Beckman Research Institute, City of Hope National Medical Center, Duarte, CA, United States; 2 School of Community & Global Health, Claremont Graduate University, Claremont, CA, United States; 3 Department of Psychological Science, Claremont McKenna College, Claremont, CA, United States; 4 School of Social Work, California State University, San Bernardino, CA, United States

**Keywords:** diabetes, health disparities, healthcare, racial and ethnic minorities, screening

## Abstract

**Background:**

Compared to non-Hispanic White adults, racial/ethnic minority adults have a higher risk of developing diabetes and its complications. Proper use of healthcare services is important for reducing the prevalence of diabetes and the risk of complications. However, minority populations tend to receive a lower quality of healthcare service and have greater barriers to diabetes self-management. This study aims to determine racial/ethnic disparities in the utilization of diabetes-related healthcare services and estimate factors that are associated with the utilization.

**Methods:**

This study used the 2016–2021 Behavioral Risk Factor Surveillance System data. Accomplishment of diabetes-related healthcare services (annual clinical care, self-care) among patients with diabetes and diabetes screening rates among adults at risk of developing diabetes were assessed. Chi-square tests explored associations between race/ethnicity and healthcare service use. Weighted logistic regression models estimated factors independently associated with utilization, controlling for covariates.

**Results:**

Patients with diabetes who reported fully completing annual clinical care exams were less than 50% for all racial and ethnic groups. Asian American patients reported the lowest percentage of fully completing self-care practices (1.2%). Compared to non-Hispanic White patients, Hispanic and Latino patients had the lowest odds of fully completing all annual exams (OR = 0.10, 95% CI, 0.07, 0.14) and self-care practices (OR = 0.63, 95% CI, 0.43, 0.92). Except for non-Hispanic Black adults, asymptomatic adults at risk from other racial and ethnic minority groups had significantly lower odds of having screening, and Asian American adults had the lowest odds of screening uptake (OR = 0.55, 95% CI, 0.52, 0.59) compared to non-Hispanic White adults. Healthcare insurance coverage, access to healthcare, self-rated general health status, and alcohol consumption were significantly associated with diabetes-related healthcare utilization status.

**Conclusions:**

Racial and ethnic disparities exist in diabetes-related healthcare service utilization. These findings underscore an urgent need to improve diabetes-related healthcare service use for racial and ethnic minority groups.

## Introduction

Over the past few decades, the prevalence of diabetes has increased steadily in the world, becoming one of the leading causes of death (1). In the United States, approximately 38 million people have diabetes, and 1 in 5 of them are undiagnosed (2). CDC National Diabetes Statistics Report indicated that the prevalence of both diagnosed and undiagnosed diabetes was higher for racial and ethnic minorities (REMs) compared to non-Hispanic White people (NHWs) (3). Although the incidence of diabetes has remained relatively stable among NHWs, the rates have increased significantly for all other REMs (4–6).

Despite efforts made to curb the rising rate of diabetes among REMs, diabetes continues to be a public health crisis. The overall medical cost of people with diagnosed diabetes is about $413 billion yearly, which is twice as high as that of individuals without diabetes (2). To decrease the medical and societal burden caused by diabetes, early detection and early treatment are essential to control diabetes conditions and reduce the risk of developing long-term complications (7–9). Evidence-based diabetes healthcare practices can prevent acute complications and further reduce costs (7–9). However, to deal with this public health crisis, various social and economic barriers (e.g., language barriers, low quality of healthcare service, etc.) that impede REMs from accessing diabetes care need to be addressed (10). Taking into consideration the increased prevalence, limited access to care, lower quality of care received, and various barriers to self-management of the disease, our public health and healthcare systems need to better understand and address the needs of REMs and improve their care.

Given the limited research on how REMs utilize diabetes-related healthcare services (DHS), this study will be one of the first attempts to evaluate disparities in their DHS utilization (including clinical care, self-care, and prevention screening) and identify factors associated with such disparities using nationally representative data. The findings of this study could inform recommendations for improving diabetes-related healthcare for REMs.

## Methods

### Data source and sample

This secondary data analysis study is based on data from the 2016–2021 Behavioral Risk Factor Surveillance System (BRFSS), a national telephone survey system that collected health-related information about the U.S. noninstitutionalized adult population (≥18 years) living in all 50 states, the District of Columbia, and three U.S. territories at the state level and local level (11). Responses of BRFSS participants are self-reported, and there is no proxy interview (12).

The sample of this study includes: 1) adults with diabetes, excluding those with pre-diabetes/borderline diabetes and gestational diabetes; 2) asymptomatic adults who are at risk of developing diabetes and should have diabetes screening tests. This study created a binary variable (yes/no) to indicate whether the respondent was diagnosed with diabetes.

Asymptomatic adults who are at risk of developing diabetes were defined based on the 2026 American Diabetes Association (ADA) Standards of Care in Diabetes (13). According to the ADA screening criteria, criteria for screening for diabetes in asymptomatic adults are: 1) adults with overweight or obesity (BMI ≥25 kg/m^2^ or ≥23 kg/m^2^ in individuals of Asian ancestry) who have one or more of the following risk factors (including first-degree relative with diabetes; high-risk race, ethnicity, and ancestry (e.g., African American, Latino, Native American, Asian American); history of cardiovascular disease; hypertension (≥130/80 mmHg or on therapy for hypertension); HDL cholesterol level <35 mg/dL (<0.9 mmol/L) and/or triglyceride level >250 mg/dL (>2.8 mmol/L); individuals with polycystic ovary syndrome; physical inactivity; and other clinical conditions associated with insulin resistance (e.g., severe obesity, acanthosis nigricans, metabolic dysfunction-associated steatotic liver disease)); 2) people with prediabetes [A1C ≥5.7% (≥39 mmol/mol), IGT, or IFG] should be tested yearly; 3) people who were diagnosed with GDM should have testing at least every 1–3 years; 4) for all other people, testing should begin at age 35 years.

Previous research found that the majority of adults who are overweight or obese have one or more risk factors mentioned in the criteria, such as hypertension; thus, screening should be considered in adults who are overweight or obese regardless of any additional risk factors (14, 15). The US Preventive Services Task Force identified being obese and overweight as the strongest risk factor for developing diabetes (16). Therefore, this study defined asymptomatic adults who are at risk of developing diabetes and should have diabetes screening tests as adults who have not been diagnosed with diabetes but are aged ≥35 years and adults who are aged <35 years with overweight and obese BMI. Adults who have been diagnosed with pre-diabetes or borderline diabetes and women with gestational diabetes were excluded from this study since they were recommended to have specific healthcare services; more details are in the Discussion section. Detailed definitions of the study population and original BRFSS questions can be found in **Online Supplementary Data Sheet 1**. However, since ADA officially lowered the recommended age for the start of Type 2 diabetes screening for adults from 45 to 35 in 2022 and this study used the most recent 2026 guideline with a dataset collected from 2016-2021, a sensitivity analysis using the previous guidelines with an age threshold of 45 years was conducted to identify any potential effects.

Given data are de-identified and publicly available (17), the standard rule exemption applied and did not require an institutional review board review. Additional information regarding descriptions, questionnaires, and sampling methodology can be found on the BRFSS website (18).

### Measures

The measures of this study were selected based on the Andersen Healthcare Utilization Model (19) and previous literature (14, 20–25). This research included two studies: 1) DHS utilization of patients with diabetes and 2) screening uptake of adults at risk of developing diabetes. The independent variable for both studies is self-reported race and ethnicity, a defined variable by BRFSS that includes non-Hispanic Asian American (AA), non-Hispanic American Indian or Alaskan Native (AIAN), Hispanic or Latinx (H/L), non-Hispanic multiracial and non-Hispanic others (MRO), non-Hispanic Black (NHB), non-Hispanic White (NHW), and non-Hispanic Pacific Islander (PI).

For study 1, outcome variables included annual clinical care utilization and self-care practice. Annual clinical care utilization (performed by health professionals) was measured by ADA recommendations (13): having ≥2 hemoglobin A1C (HbA1C) tests, ≥1 eye examination, and ≥1 foot examination. Self-care practice was assessed by engaging in any physical activity or exercise during leisure time in the past month, self-monitoring blood glucose daily, self-checking feet for sores or irritations daily, and participation in any diabetes self-management education programs. The completion status of clinical and self-care was categorized into fully completed (complete all four activities), partially completed (complete one, two, or three activities), and not completed (complete 0 activities). For study 2, the outcome variable was self-reported receipt of the diabetes screening test, measured as a binary outcome (Yes or No). This standard of accomplishment was based on the previous literature (20–25) and the aim of this study; more details are in the Discussion section.

Covariates were sociodemographic characteristics (age, sex at birth, marital status, annual household income, educational attainment, and employment status), healthcare insurance and access (insurance coverage, routine annual checkups, regular healthcare providers, and unmet healthcare needs because of cost), health status (self-rated general health status, other critical chronic conditions), and health behaviors (smoking status, alcohol consumption). Specifically, covariates of study 1 included insulin use and whether diabetes had affected eyes (as an indicator of poor diabetes control) (25); covariates of study 2 included leisure-time physical activity (22). Additionally, since this study pooled 2016–2021 data (covering the COVID-19 pandemic period from March 2020 to May 2023), the year of interview variable was also included as a covariate to explore the potential pandemic impact. Detailed codes of measurement variables and original BRFSS questions can be found in **Online Supplementary Data Sheet 1**.

### Data analysis

Descriptive statistical analyses were conducted taking into consideration the complex sample design (26). For both studies, descriptive estimates were obtained from subpopulation analyses (patients with diabetes and individuals at risk) (26). Additionally, second-order (Satterthwaite) Rao-Scott chi-square tests were used to explore associations between independent variables and outcomes. Weighted multinomial logistic regression models (for study 1) and a weighted multivariable logistic regression model (for study 2) were conducted using PROC SURVEYLOGISTIC (26). Logistic regressions provided adjusted odds ratios with all covariates in the models. Multicollinearity analysis was conducted for variables that are likely correlated, such as insurance coverage, regular provider, and routine checkups and variance inflation factors (VIFs) were reported. All analyses were weighted using the weight variables provided by the BRFSS datasets, including variables of sampling weights, stratifications, and domains (27). A p-value less than.05 was set as the test of statistical significance. All statistical analyses were performed using Statistical Analysis Software (SAS), version 9.4 (SAS Institute Inc., Cary, NC).

## Results

The total sample size of the 6-year BRFSS dataset (2016-2021) was 2,632,674, including 354,307 (13.5% of total, unweighted) patients diagnosed with diabetes and 1,990,503 (78.5% of total, unweighted) asymptomatic adults who are at risk of developing diabetes. All percentages reported in this study are weighted unless specified otherwise.

### Characteristics of patients with diabetes

Among patients with diabetes, 4.2% (N = 6,155) were AAs, 1.4% (N = 9,080) were AIANs, 18.3% (N = 30,677) were H/Ls, 1.9% (N = 10,052) were MROs, 15.8% (N = 41,184) were NHBs, 58.3% (N = 247,933) were NHWs, and 0.2% (N = 1,846) were PIs. As shown in **Table 1**, more than 75% of patients were aged over 45 in all racial and ethnic groups. Notably, 72.2% of AA patients attended or graduated from college or technical school, while 51.3% of H/L patients did not graduate high school. AA patients reported the highest household income, with 53.5% having $50,000 or more annual income; H/L patients had the lowest household income, with 30.4% reporting less than $15,000 per year. Results also indicated that most patients for all REM groups had good access to healthcare services, including health insurance, annual routine health checkups, regular healthcare providers, and affordable healthcare needs.

**Table 1:** Characteristics of patients with diabetes by racial and ethnic category, 2016–2021, BRFSS.

Race and ethnicity	AA N = 6,155	AIAN N = 9,080	H/L N = 30,677	MRO N = 10,052	NHB N = 41,184	NHW N = 247,933	PI N = 1,846	P-value ^c^
Characteristics weighted %								
Age (Years)								<.0001
*18-24*	1.8	1.4	1.8	2.0	1.3	1.1	1.7	
*25-34*	3.8	4.8	5.4	4.9	3.6	2.4	4.6	
*35-44*	14.0	11.2	12.0	10.7	9.5	5.9	18.4	
*45-54*	16.8	22.7	21.6	16.0	19.2	14.5	28.8	
*55-64*	25.7	27.2	27.4	28.7	28.8	25.7	21.2	
*≥ 65*	38.0	32.8	31.7	37.7	37.6	50.3	25.4	
Sex at Birth								<.0001
*Male*	56.3	50.4	48.7	48.3	44.4	52.0	48.3	
*Female*	43.7	49.5	51.3	51.7	55.6	48.1	51.7	
Marital Status								<.0001
*Married or Living with a partner*	69.9	47.1	56.9	49.3	40.0	58.9	59.8	
*Other*	30.1	52.9	43.1	50.7	60.0	41.1	40.2	
Educational Attainment								<.0001
*Did not graduate high school*	8.3	26.3	51.3	16.3	20.7	12.4	17.6	
*Graduated high school*	19.6	28.8	22.2	24.8	32.6	32.5	35.6	
*Attended college/technical school*	25.0	32.6	17.7	38.6	31.2	34.2	30.7	
*Graduated from college/technical school*	47.2	12.3	8.8	20.4	15.6	20.9	16.1	
Employment Status								<.0001
*Unemployed*	6.5	7.3	7.7	4.8	6.9	4.1	7.6	
*Not in the labor force*	42.9	62.2	55.8	61.6	61.4	64.1	49.9	
*Employed*	50.7	30.5	36.6	33.6	31.7	31.7	42.4	
Annual Household Income								<.0001
*Less than $15,000*	12.5	24.8	30.4	17.8	21.0	10.8	15.4	
*$15,000 to less than $25,000*	14.1	26.4	28.1	21.3	25.0	18.7	23.4	
*$25,000 to less than $35,000*	11.0	11.2	12.5	11.0	12.9	12.5	12.8	
*$35,000 to less than $50,000*	8.9	10.9	16.7	13.8	12.5	15.0	10.3	
*$50,000 or more*	53.5	26.8	18.4	36.2	28.6	42.9	38.1	
Health Insurance Coverage								<.0001
*Yes*	94.8	93.7	82.6	93.9	92.3	95.9	89.0	
Routine Annual Checkup								<.0001
*Yes*	90.6	88.8	87.3	90.3	93.6	92.4	87.2	
Regular Healthcare Provider								<.0001
*Yes*	94.5	87.7	84.7	91.3	93.5	95.5	87.6	
Unmet Healthcare Needs								<.0001
*No*	85.8	80.6	78.1	83.3	83.4	88.7	77.6	
Insulin Use								<.0001
*Yes*	22.8	35.7	32.5	33.9	36.3	32.7	32.6	
Affected Eye (Control Status)								<.0001
*Yes*	30.9	26.5	23.7	21.1	23.1	17.0	39.0	
Self-rated General Health Status								<.0001
*Poor*	9.6	19.4	18.2	16.2	12.9	13.3	12.6	
*Fair*	19.8	31.5	41.1	30.6	31.1	26.3	30.1	
*Good/Better*	70.6	49.1	40.7	53.2	56.0	60.4	57.3	
Tobacco Use								<.0001
*Current smoker*	7.5	23.7	11.7	18.5	16.5	14.7	19.0	
*Former smoker*	22.3	35.4	25.7	34.6	26.5	38.8	28.5	
*Non-smoker*	70.2	40.9	62.7	46.9	57.0	46.4	52.5	
Alcohol Consumption								<.0001
***Heavy drinker***	**1.6**	**2.9**	**2.8**	**4.1**	**2.5**	**3.0**	**2.7**	

^a^ Numbers of all race/ethnicity do not add up to the total number of patients due to missing cases.

^b^ Percentages may not add up equal to 100% due to rounding.

^c^ Determined by Second-order (Satterthwaite) Rao-Scott chi-square test.

Additionally, AA patients were less likely to use insulin (22.8%) than others. PI patients (39.0%) were more likely to have eyes affected by diabetes, which indicated the poor status of diabetes control. Approximately 1 in 5 H/L patients and AIAN patients self-evaluated that their overall health conditions were not good. AIAN patients had a higher proportion of tobacco users than others: 59.1% of them were current/former smokers. Meanwhile, MRO patients (4.1%) tend to be heavy drinkers compared to others.

### Characteristics of asymptomatic adults at risk

Study 2 included approximately two million participants who were at risk of developing diabetes. Among them, 5.0% (N = 41,547) were AAs, 0.9% (N = 29,568) were AIANs, 15.8% (N = 150,977) were H/Ls, 1.8% (N = 50,193) were MROs, 11.3% (N = 140,596) were NHBs, 65.0% (N = 1,531,102) were NHWs, and 0.2% (N = 7,754) were PIs. **Table 2** shows participants’ demographic, healthcare, and health behavior characteristics. AA asymptomatic adults reported the highest educational level, while H/L adults reported the lowest. More than half of all participants were employed, regardless of their racial group. However, H/L adults tended to have less annual household income compared to others. NHW and AA participants were more likely to have an annual household income of over $50,000.

**Table 2:** Characteristics of asymptomatic adults at risk of diabetes by racial and ethnic category, 2016–2021, BRFSS.

Race and ethnicity	AA N = 41,547	AIAN N = 29,568	H/L N = 150,977	MRO N = 50,193	NHB N = 140,596	NHW N = 1,531,102	PI N = 7,754	P-value ^c^
Characteristics weighted %								
Age (Years)								<.0001
*18-24*	11.0	7.4	10.8	9.7	8.1	5.4	11.9	
*25-34*	16.3	15.0	19.6	15.4	15.3	10.7	20.8	
*35-44*	29.6	22.2	28.2	24.5	23.8	17.7	28.1	
*45-54*	19.3	20.7	19.3	17.4	20.8	19.0	18.0	
*55-64*	13.4	18.2	12.3	15.8	16.9	20.4	13.1	
*≥ 65*	10.4	16.5	9.7	17.1	15.2	26.9	8.0	
Sex at Birth								<.0001
*Male*	53.5	50.9	52.1	51.8	46.3	48.9	51.4	
*Female*	46.5	49.1	47.9	48.2	53.7	51.1	48.6	
Marital Status								<.0001
*Married or Living with a partner*	66.1	46.7	57.0	51.3	38.6	63.9	54.7	
*Other*	33.9	53.3	43.0	48.7	61.4	36.1	45.3	
Educational Attainment								<.0001
*Did not graduate high school*	4.0	17.7	33.3	9.9	12.2	7.0	10.7	
*Graduated high school*	15.1	33.8	27.1	25.6	30.7	27.2	34.8	
*Attended college/technical school*	21.5	32.3	23.7	35.9	33.1	32.3	31.1	
*Graduated from college/technical school*	59.5	16.3	15.9	28.6	24.0	33.5	23.4	
Employment Status								<.0001
*Unemployed*	5.6	9.0	7.9	6.9	8.5	4.3	9.3	
*Not in the labor force*	25.3	37.2	28.1	33.3	30.9	37.0	21.4	
*Employed*	69.1	53.8	64.0	59.8	60.6	58.7	69.3	
Annual Household Income								<.0001
*Less than $15,000*	5.9	17.0	17.3	10.9	14.2	5.5	9.9	
*$15,000 to less than $25,000*	9.5	21.7	23.9	14.9	20.0	10.8	15.3	
*$25,000 to less than $35,000*	7.3	12.1	14.0	10.6	12.0	8.6	12.3	
*$35,000 to less than $50,000*	9.7	13.3	13.5	13.0	14.1	12.7	13.2	
*$50,000 or more*	67.5	35.9	31.3	50.5	39.7	62.3	49.2	
Health Insurance Coverage								<.0001
*Yes*	92.7	87.7	72.7	89.0	87.2	93.2	85.1	
Routine Annual Checkup								<.0001
*Yes*	69.8	69.7	65.2	69.8	80.5	74.2	70.3	
Regular Healthcare Provider								<.0001
*Yes*	79.6	71.8	63.1	75.9	78.8	83.6	73.8	
Unmet Healthcare Needs due to Cost								<.0001
*No*	91.1	82.0	80.7	83.4	84.3	90.1	84.8	
Self-rated General Health Status								<.0001
*Poor*	1.7	7.9	3.4	4.9	3.6	3.5	2.5	
*Fair*	6.1	15.7	17.7	12.6	13.9	9.8	10.7	
*Good/Better*	92.2	76.5	79.0	82.5	82.5	86.7	86.9	
Tobacco Use								<.0001
*Current smoker*	7.7	29.2	12.5	22.2	18.5	16.1	19.1	
*Former smoker*	13.1	24.8	17.4	24.4	15.3	29.6	18.8	
*Non-smoker*	79.2	46.0	70.1	53.3	66.3	54.3	62.1	
Alcohol Consumption								<.0001
*Heavy drinker*	2.5	8.0	5.4	7.0	5.3	7.7	8.6	
Leisure-time Physical Activity								<.0001
***Yes***	**82.0**	**72.6**	**69.0**	**78.7**	**71.9**	**78.3**	**78.5**	

^a^ Numbers of all race/ethnicity do not add up to the total number of patients due to missing cases.

^b^ Percentages may not add up equal to 100% due to rounding.

^c^ Determined by Second-order (Satterthwaite) Rao-Scott chi-square test.

Most participants had access to healthcare insurance, providers, and services like annual checkups. However, H/L adults reported the lowest proportions of having insurance coverage (72.7%), providers (63.1%), and annual checkups (65.2%). In particular, almost 1 in 5 H/L participants had unmet healthcare needs due to cost.

Moreover, AA participants were more likely to self-rate good/better health than others, while AIAN participants tended to feel their general health status was poor. AIAN participants were more likely to smoke: 54% of them were current/former smokers. PI adults had the highest proportion of alcohol drinkers, and H/L were less likely to be physically active.

### DHS utilization status

As shown in **Table 3**, less than 50% of patients reported fully completing annual clinical care exams for all racial and ethnic groups. Compared to NHW patients with diabetes, REM patients reported lower percentages of fully completing diabetes-related clinical care activities (NHW: 43.3% versus NHB: 43.0%, AA: 39.3%, H/L: 28.5%, AIAN: 39.9%, PI: 27.2%, and MRO: 41.9%). For self-care practice, AA patients reported the lowest percentage of full completion, while NHB patients reported the highest (1.2% versus 7.1%). Among all asymptomatic adults who are at risk of developing diabetes, MRO adults (56.7%) and AA adults (55.3%) were more likely to have diabetes screening tests. NHB adults had a smaller proportion of taking screening: only 38.9% of them had a test within the past three years.

**Table 3:** Diabetes-related healthcare service use (clinical care, self-care, screening) by racial and ethnic category, 2016–2021, BRFSS.

Race and ethnicity	AA	AIAN	H/L	MRO	NHB	NHW	PI	P-value^c^
Characteristics N, weighted %								
Study 1 Diabetes Clinical Care								<.0001
*Fully Completed*	510, 39.3	1489, 39.9	3337, 28.5	1253, 41.9	8513, 43.0	38703, 43.3	226, 27.2	
*Partially Completed*	781, 60.0	2008, 59.6	7178, 68.3	1693, 57.3	10786, 56.6	46417, 56.3	397, 70.7	
*Not Completed*	14, 0.7	20, 0.5	235, 3.2	25, 0.8	86, 0.5	273, 0.5	19, 2.0	
Diabetes Self-care								<.0001
*Fully Completed*	99, 1.2	600, 5.6	1233, 3.3	495, 4.0	3228, 7.1	11701, 4.9	72, 2.6	
*Partially Completed*	5933, 98.5	8320, 93.9	28878, 96.1	9399, 95.5	37374, 92.5	232923, 94.6	1734, 96.9	
*Not Completed*	18, 0.3	37, 0.5	165, 0.6	46, 0.4	166, 0.4	1112, 0.5	19, 0.5	
Study 2 Diabetes Screening Test								<.0001
*Yes*	11227,44.7	9907,57.1	44194,57.6	15748,56.7	51194, 61.1	447870,60.0	3134,51.0	
***No***	**11979,55.3**	**6981,42.9**	**27584,42.4**	**10657,43.3**	**28419,38.9**	**268705,40.0**	**2688,49.0**	

^a^ Numbers of all race/ethnicity do not add up to the total number of patients due to missing cases.

^b^ Percentages may not add up equal to 100% due to rounding.

^c^ Determined by Second-order (Satterthwaite) Rao-Scott chi-square test.

### Racial and ethnical disparities in DHS utilization

A significant association was found between race and ethnicity and DHS utilization. Based on the results of Rao-Scott Chi-square tests, race and ethnicity are significantly related to clinical healthcare service utilization (χ^2R-S^=142.0, DF = 3.7, p<0.0001), self-care practice accomplishment (χ^2R-S^=208.2, DF = 7.7, p<0.0001), and diabetes screening test uptake (χ^2R-S^=490.2, DF = 4.3, p<0.0001). **Tables 4**–**6** present the results of logistic regression models.

**Table 4:** Results of study 1, DHS utilization (clinical care) among patients with diabetes.

Factors	Study 1 diabetes-related annual clinical care activities fully completed			Study 1 diabetes-related annual clinical care activities partially completed		
	OR	95% CI	p-value	OR	95% CI	p-value
Race/Ethnicity			<.0001			<.0001
*AA*	0.61	0.24, 1.57		0.72	0.28, 1.85	
*AIAN*	0.84	0.41, 1.70		0.96	0.47, 1.95	
*H/L*	0.10	0.07, 0.14		0.18	0.13, 0.25	
*MRO*	0.56	0.31, 1.01		0.59	0.33, 1.05	
*NHB*	0.99	0.68, 1.43		1.00	0.67, 1.45	
*PI*	0.15	0.07, 0.29		0.29	0.15 0.57	
NHW (Ref)						
Health Insurance Coverage			0.01			0.01
*Yes*	2.36	1.03, 5.41		1.73	0.77, 3.88	
No (Ref)						
Regular Healthcare Provider			<.0001			<.0001
*Yes*	5.95	2.86, 12.37		3.12	1.54, 6.33	
No (Ref)						
Routine Checkup			<.0001			<.0001
*Yes, within the past year*	7.01	1.33, 36.94		2.50	0.50, 12.36	
Never (Ref)						
Insulin Use			<.0001			<.0001
*Yes*	3.04	1.45, 6.38		1.52	0.73, 3.17	
No (Ref)						
Affected Eye (Control Status)			0.00			0.00
*Yes*	3.44	1.51, 7.82		2.70	1.19, 6.11	
No (Ref)						
Self-rated General Health Status			0.00			0.00
*Fair*	2.17	1.07, 4.38		2.42	1.21, 4.85	
Good/Better (Ref)						
Diagnosis with Arthritis			0.00			0.00
*Yes*	2.55	1.42, 4.58		2.25	1.26, 4.01	
No (Ref)						
Alcohol Consumption-Heavy Drinker			<.0001			<.0001
*Yes*	0.09	0.04, 0.20		0.11	0.05, 0.23	
*No (Ref)*						

^a^ Race/ethnicity: non-Hispanic Asian American (AA), non-Hispanic American Indian or Alaskan Native (AIAN), Hispanic or Latinx (H/L), non-Hispanic multiracial and non-Hispanic others (MRO), non-Hispanic Black (NHB), non-Hispanic White (NHW), and non-Hispanic Pacific Islander (PI).

^b^ AOR, adjusted odds ratio; CI, confidence interval

^c^ AORs were obtained from multinomial logistic regression models

^d^ Covariates were sociodemographic characteristics (age, sex at birth, marital status, annual household income, educational attainment, and employment status), healthcare insurance and access (insurance coverage, routine annual checkups, regular healthcare providers, and unmet healthcare needs because of cost), health status (self-rated general health status, other critical chronic conditions), and health behaviors (smoking status, alcohol consumption), insulin use, and whether diabetes had affected eyes)

**Table 5:** Results of study 1, DHS utilization (self-care) among patients with diabetes.

Factors	Study 1 diabetes-related self-care practice activities fully completed			Study 1 diabetes-related self-care practice activities partially completed		
	AOR	95% CI	p-value	AOR	95% CI	p-value
Race/Ethnicity			<.0001			<.0001
*AA*	0.43	0.15, 1.26		2.19	0.79, 6.04	
*AIAN*	1.23	0.65, 2.32		0.70	0.39, 1.25	
*H/L*	0.63	0.43, 0.92		0.81	0.57, 1.15	
*MRO*	0.97	0.60, 1.58		1.13	0.72, 1.77	
*NHB*	1.77	1.23, 2.54		0.84	0.59, 1.20	
*PI*	0.52	0.21, 1.29		0.86	0.38, 1.95	
NHW (Ref)						
Health Insurance Coverage			0.01			0.01
*Yes*	2.89	1.51, 5.51		1.69	1.03, 2.77	
No (Ref)						
Regular Healthcare Provider			0.00			0.00
*Yes*	2.88	1.53, 5.41		2.62	1.64, 4.21	
No (Ref)						
Insulin Use			<.0001			<.0001
*Yes*	19.97	10.26, 38.88		8.14	4.24, 15.62	
No (Ref)						
Affected Eye (Control Status)			0.00			0.00
*Yes*	3.06	1.75, 5.33		2.44	1.44, 4.14	
No (Ref)						
Self-rated General Health Status			0.00			0.00
*Poor*	0.48	0.26, 0.89		0.69	0.39, 1.20	
*Fair*	0.43	0.27, 0.69		0.52	0.34, 0.81	
Good/Better (Ref)						
Alcohol Consumption-Heavy Drinker			<.0001			<.0001
*Yes*	0.20	0.09, 0.42		0.38	0.19, 0.74	
*No (Ref)*						

^a^ Race/ethnicity: non-Hispanic Asian American (AA), non-Hispanic American Indian or Alaskan Native (AIAN), Hispanic or Latinx (H/L), non-Hispanic multiracial and non-Hispanic others (MRO), non-Hispanic Black (NHB), non-Hispanic White (NHW), and non-Hispanic Pacific Islander (PI).

^b^ AOR, adjusted odds ratio; CI, confidence interval

^c^ AORs were obtained from multinomial logistic regression models

^d^ Covariates were sociodemographic characteristics (age, sex at birth, marital status, annual household income, educational attainment, and employment status), healthcare insurance and access (insurance coverage, routine annual checkups, regular healthcare providers, and unmet healthcare needs because of cost), health status (self-rated general health status, other critical chronic conditions), and health behaviors (smoking status, alcohol consumption), insulin use, and whether diabetes had affected eyes)

**Table 6:** Results of study 2, DHS utilization (screening tests) among asymptomatic adults.

Factors	Study 2 diabetes screening service ever taken a screening test within the past 3 years		
	OR	95% CI	p-value
Race/Ethnicity			<.0001
*AA*	0.55	0.52, 0.59	
*AIAN*	0.92	0.86, 0.98	
*H/L*	0.91	0.89, 0.94	
*MRO*	0.86	0.82, 0.90	
*NHB*	1.03	1.00, 1.06	
*PI*	0.71	0.64, 0.79	
NHW (Ref)			
Age (Years)			<.0001
*18-24*	0.29	0.27, 0.31	
*25-34*	0.52	0.49, 0.56	
*35-44*	0.69	0.65, 0.73	
*45-54*	0.87	0.82, 0.92	
*55-64*	1.07	1.02, 1.13	
*≥ 65 (Ref)*			
Gender			0.01
*Male*	0.96	0.93, 0.99	
*Female (Ref)*			
Marital Status			<.0001
*Married or Living with a partner*	1.18	1.14, 1.22	
Other (Ref)			
Educational Attainment			<.0001
*Graduated from college or technical school*	1.46	1.33, 1.61	
*Attended college or technical school*	1.41	1.31, 1.52	
*Graduated high school*	1.20	1.12, 1.29	
*Did not graduate high school (Ref)*			
Annual Household Income			<.0001
*$15,000 to less than $25,000*	1.19	1.11, 1.26	
*$25,000 to less than $35,000*	1.21	1.12, 1.30	
*$35,000 to less than $50,000*	1.29	1.11, 1.28	
*$50,000 or more*	1.23	1.15, 1.31	
*Less than $15,000 (Ref)*			
Health Insurance Coverage			<.0001
*Yes*	1.25	1.18, 1.33	
No (Ref)			
Regular Healthcare Provider			<.0001
*Yes*	1.52	1.45, 1.59	
No (Ref)			
Routine Checkup			<.0001
*Yes, within the past year*	3.36	2.78, 4.07	
*Yes, but not within the past year*	1.62	1.34, 1.96	
Never (Ref)			
Self-rated General Health Status			<.0001
*Fair*	1.12	1.07, 1.18	
*Good/Better (Ref)*			
Diagnosis with Heart Disease			<.0001
*Yes*	1.26	1.16, 1.37	
No (Ref)			
Diagnosis with Asthma			0.01
*Yes*	1.06	1.01, 1.10	
No (Ref)			
Diagnosis with Kidney Disease			0.001
*Yes*	1.17	1.06, 1.28	
*No (Ref)*			
Diagnosis with Arthritis			<.0001
*Yes*	1.33	1.28, 1.39	
No (Ref)			
Diagnosis with Depressive Disorder			<.0001
*Yes*	1.19	1.14, 1.23	
*No (Ref)*			
Leisure-time Physical Activity			<.0001
*No*	0.88	0.85, 0.92	
Yes (Ref)			
Alcohol Consumption-Heavy Drinker			<.0001
*Yes*	0.86	0.81, 0.91	
No (Ref)			
Interview Year			<.0001
*2021*	0.67	0.58, 0.78	
*2020*	0.74	0.70, 0.77	
*2016 (Ref)*			

^a^ Race/ethnicity: non-Hispanic Asian American (AA), non-Hispanic American Indian or Alaskan Native (AIAN), Hispanic or Latinx (H/L), non-Hispanic multiracial and non-Hispanic others (MRO), non-Hispanic Black (NHB), non-Hispanic White (NHW), and non-Hispanic Pacific Islander (PI).

^b^ AOR, adjusted odds ratio; CI, confidence interval

^c^ AORs were obtained from multivariable logistic regression models

^d^ Covariates were sociodemographic characteristics (age, sex at birth, marital status, annual household income, educational attainment, and employment status), healthcare insurance and access (insurance coverage, routine annual checkups, regular healthcare providers, and unmet healthcare needs because of cost), health status (self-rated general health status, other critical chronic conditions), and health behaviors (smoking status, alcohol consumption, leisure-time physical activity).

For clinical DHS, compared to NHW patients, H/L patients had 90 percent lower odds of fully completing all annual exams (OR = 0.10, 95% CI, 0.07, 0.14) and 82 percent lower odds of partially completing annual exams (OR = 0.18, 95% CI, 0.13, 0.25), respectively. Besides, PI patients had lower odds of achieving all clinical healthcare activities (OR = 0.15, 95% CI, 0.07, 0.29) as well as partially achieving (OR = 0.29, 95% CI, 0.15, 0.57).

Additionally, H/L patients with diabetes had 37 percent lower odds of fully completing all self-care practices (OR = 0.63, 95% CI, 0.43, 0.92) compared to NHW patients. Notably, NHB patients had 77 percent greater odds of fully achieving self-care activities than NHW patients (OR = 1.77, 95% CI, 1.23, 2.54).

Regarding diabetes screening test uptake, except NHBs, other REM asymptomatic adults at risk were significantly less likely to report receiving screening. Compared to NHW adults who are at risk of developing diabetes, AAs had the lowest odds of screening uptake (OR = 0.55, 95% CI, 0.52, 0.59), followed by PIs (OR = 0.71, 95% CI, 0.64, 0.79), MROs (OR = 0.86, 95% CI, 0.82, 0.90), H/Ls (OR = 0.91, 95% CI, 0.89, 0.94), and AIANs (OR = 0.92, 95% CI, 0.86, 0.98). NHB adults had greater odds of taking diabetes screening than NHW adults, with an OR of 1.03 (95% CI, 1.00, 1.06). Additionally, in sensitivity analyses, we found that the results of racial/ethnic disparities in diabetes screening did not change substantially when we used the 2021 ADA guideline with an age threshold of 45 years for screening. Details can be found in **Online Supplementary Data Sheet 1**.

### Factors associated with fully accomplishing DHS utilization

The final multinomial logistic regression models (for study 1) and multivariable logistic regression model (for study 2) indicate that having healthcare insurance and regular providers were significantly associated with all types of DHS utilization. Self-rated general health status and alcohol consumption also had significant associations with the achievement of three services. **Tables 4**–**6** showed the results from the logistic regression models and all ORs were adjusted. As shown in **Tables 4**–**6**, respondents who had healthcare insurance had 189 percent (OR of 2.89 with 95% CI, 1.51, 5.51), 136 percent (OR of 2.36 with 95% CI, 1.03, 5.41), and 25 percent (OR of 1.25 with 95% CI, 1.18, 1.33) greater odds to fully complete self-care practice, clinical care activities, and screening test, respectively. Having a regular healthcare provider also associated with achieving all self-care practices by 188 percent (OR of 2.88 with 95% CI, 1.53, 5.41), fully completing annual clinical care by 495 percent (OR of 5.95 with 95% CI, 2.86, 12.37), and taking screening by 52 percent (OR of 1.52 with 95% CI, 1.45, 1.59). Patients who had a routine checkup within the past year also had a 601 percent (OR of 7.01 with 95% CI, 1.33, 36.94) greater odds of fully completing clinical care activities.

VIF values for insurance coverage, regular provider, and routine checkups are: 1) for self-care practice, insurance coverage 1.12, regular provider 1.25, routine checkups 1.19; 2) for clinical care activities, insurance coverage 1.07, regular provider 1.09, routine checkups 1.05; 3) for screening, insurance coverage 1.13, regular provider 1.26, routine checkups 1.20. All VIFs indicated moderate correlations, which are acceptable for regression models (26).

Specifically, insulin use and affected eye were also significantly associated with fully completing clinical care activities (insulin use OR of 3.04 with 95% CI, 1.45, 6.38; affected eye OR of 3.44 with 95% CI, 1.51, 7.82) and self-care activities (insulin use OR of 19.97 with 95% CI, 10.26, 38.88; affected eye OR of 3.06 with 95% CI, 1.75, 5.33). Patients who were diagnosed with arthritis also had a 155 percent (OR of 2.55 with 95% CI, 1.42, 4.58) greater odds of fully completing clinical care activities. Meanwhile, a diagnosis of other diseases was also significantly associated with greater odds of having screening tests (heart disease OR of 1.26 with 95% CI, 1.16, 1.37; asthma OR of 1.06 with 95% CI, 1.01, 1.10; kidney disease OR of 1.17 with 95% CI, 1.06, 1.28; arthritis OR of 1.33 with 95% CI, 1.28, 1.39; depressive disorder OR of 1.19 with 95% CI, 1.14, 1.23).

Moreover, participants who rated their health status as poor had lower odds to complete self-care activities (OR = 0.48, 95% CI, 0.26, 0.89); participants who rated their health status as fair had greater odds of having screening tests (OR = 1.12, 95% CI, 1.07, 1.18) and fully completing clinical care activities (OR = 2.17, 95% CI, 1.07, 4.38) but lower odds to complete self-care activities (OR = 0.43, 95% CI, 0.27, 0.69). Regarding health behaviors, heavy drinkers had significantly lower odds of using all types of service with an OR of 0.20 (95% CI, 0.09, 0.42) for self-care, an OR of 0.09 (95% CI, 0.04, 0.20) for clinical care, and an OR of 0.86 (95% CI, 0.81, 0.91) for screening. Adults who lacked leisure-time physical activities had significantly lower odds for screening (OR = 0.88, 95% CI, 0.85, 0.92).

The year of interview is not significantly associated with DHS utilization among diabetes patients on their clinical care and self-care, which indicates no statistically significant associations of the COVID-19 pandemic on them. However, it is significantly related to diabetes screening among adults at risk (OR = 0.74, 95% CI, 0.70, 0.77 for 2020; OR = 0.67, 95% CI, 0.58, 0.78 for 2021), which indicates adults at risk were significantly less likely to have screening tests during the pandemic period. Details can be found in **Online Supplementary Data Sheet 1**.

## Discussion

These two secondary analysis studies demonstrate that race and ethnicity were significantly associated with diabetes-related healthcare service (DHS) utilization. Findings suggest that racial and ethnic disparities in DHS use (including clinical care, self-care, and screening care) exist. The overall percentages of participants who had achieved all types of DHS utilization were relatively low, especially for those from some racial and ethnic minority (REM) groups. Compared to NHW patients with diabetes, H/L patients and PI patients had lower odds of completing all recommended clinical exams, and H/L patients had lower odds of achieving all self-care practices. Except for NHBs, other REM asymptomatic adults had lower odds of having diabetes screening tests. Results highlighted healthcare insurance coverage, access to healthcare providers, self-rated general health status, and alcohol consumption as significant factors that are associated with service utilization.

To our knowledge, limited studies examined diabetes-related healthcare utilization at the national level (8, 24, 25). Few studies covered healthcare services for both patients diagnosed with diabetes and asymptomatic adults who should take diabetes screening; most of the previous studies only analyzed one type of utilization for a certain population (clinical service, self-care, or screening) (14, 22–25, 28, 29). Some studies, such as Chandler RF et al. (2015) and Kamat S et al. (2019), were conducted more than 5 years ago, and the data they used were not able to reflect recent trends (28, 29). Therefore, this study presents the first nationally representative estimates of DHS utilization stratified by race and ethnicity and investigates potential factors associated with this utilization. The findings of this research can provide a comprehensive understanding of what disparities there are in this country. We thus recommend an implementation and dissemination science approach to create strategies for adopting, implementing, sustaining, and disseminating racial and ethnic tailored education programs (such as tailoring culture and language for specific race/ethnicity to improve their healthcare access, health literacy, diabetes awareness, etc.) to improve DHS utilization among REMs.

We found H/L patients reported the lowest odds ratio for annual clinical care and self-care accomplishment. Compared to others, they were less educated, had lower household incomes, were without healthcare insurance, and had worse general health. A lack of education may present challenges in understanding healthcare service instructions and less awareness of how to utilize and access services (30–33). Their low income and limited access to health care decreased DHS utilization as well (34, 35). Future healthcare policies should focus more on helping H/L populations improve insurance coverage and provide affordable healthcare services.

Despite having the highest educational and income levels, asymptomatic AA adults were least likely to be screened for diabetes compared to all others. One possible explanation is AAs were disproportionally more likely to report good health (AAs: 92.2% vs NHWs: 86.7%), though them being qualified as “at risk of developing diabetes” according to ADA guidelines. Research demonstrates that those who believe they are healthy are less likely to be screened for diabetes (36, 37), which can cause a lack of awareness of this elevated risk among AA. Also, lack of recommendations from their providers may be another barrier: providers may not be aware of the lower BMI threshold for AA, leading to fewer requests for testing (38). Primary care teams should have basic training in knowledge and skills to identify and manage diabetes. For healthcare professionals who work in general hospitals and already have the essential knowledge to identify other health needs, they should be able to refer patients to specialized diabetes doctors, which can help patients to access related diabetes care services or encourage them to have screening tests. Additionally, the language barrier can be another reason for the low rate of self-care completion. Some self-care activities (such as self-monitoring blood sugar levels) require basic training, most of which were often offered in English only, making it difficult for linguistic minority groups to take advantage of those training programs. Fluency in English and familiarity with the U.S. healthcare environment can be additional factors that related to service utilization, which may call for better navigation support for healthcare access (38–40). Our future studies will focus on the AA population and develop a culturally tailored survey to identify barriers (such as provider unawareness of lower BMI thresholds for Asian Americans and language barriers driving low self-care completion) to diabetes-related healthcare service utilization among this population.

This study also found that PI diabetes patients had the lowest percentage fully completing all clinical healthcare activities (PIs: 27.2% vs NHWs: 43.3%) and lower odds of achieving all clinical healthcare activities. PIs in this study reported lower socioeconomic status than NHWs, which is a barrier to healthcare service access. Other potential barriers include low health literacy, cultural difference and language barriers, especially for Micronesian PIs (40, 41). Additionally, literature showed that AIANs face similar barriers, such as low income/educational level and low health literacy (42–44), to accessing diabetes-related healthcare services, which can explain the reasons that AIANs in this study had lower percentages of fully complete clinical care activities and screening.

On the other hand, the results of this study indicated NHBs tend to have better percentages of completing clinical care activities (second highest, 43.0%), self-care practices (highest, 7.1%), and screening (highest, 61.1%) than other REMs. NHB adults also had greater odds of taking diabetes screening than NHW adults, with an OR of 1.03 (95% CI, 1.00, 1.06) and NHB patients had 77 percent greater odds of fully achieving self-care activities than NHW patients (OR = 1.77, 95% CI, 1.23, 2.54). These patterns were related to the increasing implementation of diabetes educational and engagement interventions targeted at NHB communities (45–47). Evidence-based community-engagement can significantly increase awareness of diabetes care and prevention, which benefits NHBs and encourages their accomplishments on DHS utilization. Although the 95%CI of NHB patients touches the borderline, we believe it is worth including and discussing. Racial and ethnic differences in DHS utilization are complex. Policymakers and practitioners should apply a targeted approach, considering culture, traditions, and specific factors associated with these differences. The patterns among NHBs set a good example for all other REMs regarding increased DHS utilization.

Also, we found that diagnoses with other diseases were associated with higher odds of completing diabetes clinical care and having regular screening tests, which can be a proxy for healthcare system engagement (i.e., patients with multiple chronic conditions visit providers more often). Other unhealthy behaviors, such as too much alcohol consumption and lack of physical activity, were significantly related to lower odds of diabetes screening, which suggests the importance of developing comprehensive interventions to promote healthy behaviors for preventing diabetes.

This study also found that people were less likely to have a screening test during the COVID-19 pandemic period, which aligns with the previous literature (48–50). The decrease in diabetes screening may be caused by system-level delays, such as the closure of hospitals and clinics. Since the screening tests require in-person clinical examination and laboratory evaluations, people may lose access to the screening. Also, to avoid the risk of potential exposure to COVID-19, many adults at risk chose to delay screening. Financial barriers caused by the pandemic lockdown also contributed to a delay in care.

The main aim of this study is to have a better understanding of racial/ethnic disparities in diabetes-related healthcare service utilization. Therefore, the comparisons focused more on broad accomplishments in clinical care and self-care practices. This study defined diabetes-related healthcare service utilization accomplishments into 3 categories (fully completed, partially completed, and not completed) based on the study aim and previous literature (20–25) to obtain an overall comparison and investigation of potential factors associated with the accomplishments. Regardless of how many activities were completed, they failed to accomplish the goal based on ADA’s recommendation, which is fully accomplishments. Thus, this study investigated factors associated with fully accomplishing DHS utilization. However, for those who partially completed one, two, three, or four clinical care and self-care practices, they may face different barriers that resulted in this difference. We will conduct a study that only focuses on adults who partially completed the clinical care and self-care practices to explore potential barriers and factors associated with their healthcare service utilization in the future.

Prediabetes is another serious health condition that is also related to hyperglycemia. Due to the failure of early diabetes control, undiagnosed diabetes adults were more likely to develop related complications than those with prediabetes, which would add a further burden (7). However, adults with prediabetes or borderline diabetes were not the study population in this study. ADA suggested that prediabetes status only indicated the increased risk for diabetes and heart disease but should not be treated as a clinical entity (13). Therefore, diabetes-related clinical care services may not be proper for adults with prediabetes or borderline diabetes. Current BRFSS questionnaires do not collect any information about diabetes-related clinical service among adults with prediabetes (11, 12, 18). Additionally, the 2017 National Standards for Diabetes Self-Management Education and Support addressed self-care management education and support (DSMES) programs and National Diabetes Prevention Program (National DPP) lifestyle change programs are tailored for different participants with different needs and expectations; DPP is designed to provide more specific skills for prediabetes while DSMES works better for patients with diabetes (51). Yet, no information about DPP was captured in BRFSS (11, 12, 18). In addition, based on ADA guidelines, for adults with prediabetes, an annual screening test is recommended (13). However, due to time and funding limitations, adults with prediabetes or borderline diabetes were not included in Study 1 or Study 2. We will conduct a study that only focuses on adults with prediabetes or borderline diabetes and measures their specific medical service utilizations in the future.

### Limitations

This study has several limitations. First, there is no racial and ethnic subgroup (Chinese, Japanese, Caribbean Black, etc.) information in the BRFSS data. Racial and ethnic subgroups were heterogeneous and had different factors that were associated with the patterns of DHS utilization. Second, all responses in the BRFSS data were self-reported and consequently subject to retrospective memory biases and lack of awareness. Another limitation is the lack of information about the duration of diagnosis and the type of diabetes. Previous research indicated that patients with different lengths of time (i.e., years) since diabetes diagnosis were significantly different in the completion of diabetes self-management (52). BRFSS did not include a question about the duration of the diagnosis. Future research should address these issues to further our understanding of this complex problem. In addition, the sample size of clinical service utilization dropped about 60% because some states did not use the optional diabetes module in the BRFSS questionnaire and many patients did not respond to the clinical service utilization questions; this limitation was caused by the design of BRFSS. However, both the clinical service utilization model and the self-care utilization model remain acceptable sample sizes. Also, the screening variable in the BRFSS questionnaire asked about tests “within the past three years”; pooling six years of data (2016–2021) with a three-year recall window creates substantial temporal overlap. This measurement issue is a limitation, as the year-of-interview covariate is insufficient control for it. Additionally, we will focus more on identifying what factors have a dominant association in diabetes-related service use and what potential relations among factors are in the future study and will conduct more analyses (such as collinearity tests) as the next step.

## Conclusions

These secondary data analyses demonstrated that race and ethnic minority status were significantly associated with DHS utilization. We found evidence for racial and ethnic disparities in diabetes-related clinical, self-care, and screening healthcare service utilization. The overall utilization of all DHS was low, which indicates that a lot of people were not getting the care they needed. Compared to NHW patients with diabetes, H/L patients and PI patients had lower odds of completing all recommended clinical exams; H/L patients had lower odds of achieving all self-care practices. Except for NHBs, other REM asymptomatic adults who were at risk for developing diabetes had lower odds of having diabetes screening tests, with AAs evidencing the lowest likelihood. Results highlight that having healthcare insurance coverage and access to healthcare providers are associated with achieving suitable care practices. Self-rated general health status and alcohol consumption were also factors that significantly associated with service utilization. Culture and language tailored education and psychoeducation programs to engage race and ethnic minorities improve healthcare access, diabetes awareness, health literacy, etc., are essential in diabetes management and care.

## Data Availability

The original contributions presented in the study are included in the article/**Supplementary Material**. Further inquiries can be directed to the corresponding authors.
