# Supplementary material for: Racial and ethnic disparities in diabetes-related healthcare service use
Source: Front Clin Diabetes Healthc. 2026 May 18;7:1675970. doi: 10.3389/fcdhc.2026.1675970 (PMC13223140; doi:10.3389/fcdhc.2026.1675970)
Supplement: Supplementary file 1 [file DataSheet1.docx]

**Online Supplement 1: Racial and Ethnic Disparities in Diabetes-Related Healthcare Service Use**

1. Measures of Study 1: Diabetes-related Healthcare Service Use for Patients
   1. Study population: Adults with diabetes, excluding adults with pre-diabetes/borderline diabetes and gestational diabetes (BRFSS questionnaire question: “Has a doctor, nurse, or other health professionals ever told you that you had diabetes?”).^1^ This study created a binary variable (yes/no) to identify whether the respondent was diagnosed with diabetes.
   2. Independent variable: Self-reported race and ethnicity. This study used a defined variable by BRFSS.^2^ The original variable has 8 categories; this study combined non-Hispanic other race and non-Hispanic Multiracial race as one group and combined the non-Mexican Hispanic and Mexican groups as Hispanic/Latino. The final categories of race/ethnicity are non-Hispanic White, non-Hispanic Black, non-Hispanic Asian American (including Asian Indian, Chinese, Filipino, Japanese, Korean, Vietnamese, and other Asian), Hispanic or Latino (including Mexican, Mexican American, Chicano/a, Puerto Rican, Cuban, other Hispanic, Latino/a, or Spanish origins), non-Hispanic American Indian or Alaskan Native, non-Hispanic Pacific Islander (including Native Hawaiian, Guamanian or Chamorro, Samoan, and other Pacific Islander), and non-Hispanic multiracial and non-Hispanic others.
   3. Dependent variable: Diabetes-related healthcare service use. This study assessed two categories for the service use status, which are annual clinical care utilization and self-care practice.
      1. Annual clinical care utilization: This study created three binary variables to determine the completion of annual clinical diabetes care in the past 12 months, including having ≥2 hemoglobin A1C tests by health professionals (yes/no), ≥1 eye examination by health professionals (yes/no), and ≥1 foot examination by health professionals (yes/no). Binary variables were recoded based on BRFSS questions “About how many times in the past 12 months has a doctor, nurse, or other health professional checked you for A-one-C?”, “When was the last time you had an eye exam in which the pupils were dilated, making you temporarily sensitive to bright light?”, and “About how many times in the past 12 months has a health professional checked your feet for any sores or irritations?”.^1^ The completion status of annual clinical diabetes care use included three categories: fully completed (complete all three activities), partially completed (complete one or two activities), not completed (complete 0 activity).
      2. Self-care practice: This study created 4 binary variables to determine the completion of self-care practices, including engaging in any physical activity or exercise during leisure time in the past month (yes/no), self-monitoring blood glucose daily (yes/no), self-checking feet for sores or irritations daily (yes/no), and ever participating in any diabetes self-management education programs (yes/no). Specifically, in the BRFSS questionnaire, self-management education programs refer to the broad category as any classes or courses that provide diabetes management knowledge.^1^ Binary variables were recoded based on BRFSS questions “During the past month, other than your regular job, did you participate in any physical activities or exercises such as running, calisthenics, golf, gardening, or walking for exercise?”, “About how often do you check your blood for glucose or sugar?”, “Including times when checked by a family member or friend, about how often do you check your feet for any sores or irritations?”, and “Have you ever taken a course or class in how to manage your diabetes yourself?”.^1^ The completion status of self-care activities included three categories: fully completed (complete all four activities), partially completed (complete one or two or three activities), not completed (complete 0 activity).
   4. Covariates
      1. Demographic variables:
         1. Age: this project used a calculated variable in BRFSS. Age groups were divided into 6 groups including ≥65, 55-64, 45-54, 35-44, 25-34, and 18-24.
         2. Sex at birth: male, female.
         3. Marital status: this study created a binary variable including two categories, which were married or living with a partner and other (divorced, widowed, separated, or never married).
         4. Annual household income: this study used a defined variable in BRFSS.^2^ Income was divided into 5 groups including less than $15,000, $15,000 to less than $25,000, $25,000 to less than $35,000, $35,000 to less than $50,000, $50,000 or more.
         5. Educational attainment: this study used a defined variable in BRFSS.^2^ Educational levels were divided into 4 groups including did not graduate high school, graduated high school, attended college or technical school, graduated from college or technical school.
         6. Employment status: this study created a three-categories variable including unemployed (out of work for 1 year or more, out of work for less than 1 year), not in the labor force (a homemaker, a student, retired, unable to work), and employed (employed for wages, self-employed).
      2. Healthcare status:
         1. Health insurance coverage: this study created a binary variable to assess coverage by health insurance (survey question: “Do you have any kind of health care coverage, including health insurance, prepaid plans such as HMOs, or government plans such as Medicare, or Indian Health Service?”).^1^
         2. Whether having a routine annual checkup in the past 12 months: this study created a three-category variable including never, not within past year, and yes within past year (About how long has it been since you last visited a doctor for a routine checkup?”).^1^
         3. Whether having a regular health care provider: this study created a binary variable (yes and no) to assess (survey question: “Do you have one person you think of as your personal doctor or health care provider?”).^1^
         4. Unmet healthcare needs because of cost: this study created a binary variable (yes and no) to assess healthcare needs (survey question: “Was there a time in the past 12 months when you needed to see a doctor but could not because of cost?”).^1^
      3. General health status:
         1. Self-rated general health status: this study created a three-category variable including poor, fair, good or better health to assess a participant’s self-rated general health (survey question: “Would you say that in general your health is…”).^1^
         2. Diagnosis with other key chronic health conditions (heart attack, heart disease, stroke, asthma, cancers except for skin cancer, chronic obstructive pulmonary disease COPD, arthritis, kidney disease, depressive disorder): this study created binary variables to assess the diagnosis status, yes and no. ^1^
      4. Insulin use: created a binary variable to assess, yes and no.
      5. Whether diabetes has affected eyes: created a binary variable to assess, yes and no (survey question: “Has a doctor ever told you that diabetes has affected your eyes or that you have retinopathy”).^1^
      6. Health behaviors:
         1. Smoking status: this study used a defined variable in BRFSS which was a three-category variable including current smoker (every day smoker, someday smoker), former smoker, and non-smoker (never smoked).^2^
         2. Alcohol consumption: this study used a defined variable in BRFSS to assess whether the participant was a heavy drinker. ^2^ It was a binary variable, yes and no.
      7. Interview year: 2016, 2017, 2018, 2019, 2020, and 2021
2. Measures of Study 2: Screening Uptake of Adults at Risk of Developing Diabetes
   1. Study population: Adults who are not diagnosed with diabetes but should be screened for type 2 diabetes. This study defined adults who are at risk of developing diabetes as adults who are aged ≥35 years and aged <35 years with overweight and obese BMI. Adults who have been diagnosed with pre-diabetes or borderline diabetes and women with gestational diabetes were excluded.
   2. Independent variable: self-reported race and ethnicity, variable categories were same as Study 1.
   3. Dependent variable: self-reported receipt of the diabetes screening test. This project created a binary variable to analyze whether respondents had the screening test (yes and no) based on the BRFSS question “Have you had a test for high blood sugar or diabetes within the past three years?”. ^1^
   4. Covariates
      1. Demographic variables: age, sex at birth, marital status, annual household income, educational attainment, and employment status. Variables categories were same as Study 1.
      2. Healthcare status: health insurance coverage, whether having a routine annual checkup in the past 12 months, whether having a regular health care provider, and if having unmet healthcare needs because of cost. Variables categories were same as Study 1.
      3. General health status: self-rated general health status and other key chronic conditions. Variables categories were same as Study 1.
      4. Health behaviors: smoking status, alcohol consumption, and leisure-time physical activity. Variables categories were same as Study 1. Study 2 also include leisure-time physical activity as a covariate, which was a calculated binary variable in BRFSS, yes and no.^1^
      5. Interview year: 2016, 2017, 2018, 2019, 2020, and 2021
3. States used the optional Diabetes Module of the BRFSS questionnaire

| **State** | **2016** | **2017** | **2018** | **2019** | **2020** | **2021** | **State** | **2016** | **2017** | **2018** | **2019** | **2020** | **2021** |
| --- | --- | --- | --- | --- | --- | --- | --- | --- | --- | --- | --- | --- | --- |
| Alabama | X | X | X | X |  |  | Montana |  | X |  | X |  | X |
| Alaska |  | X |  | X |  | X | Nebraska |  |  |  |  |  |  |
| Arizona |  | X | X |  |  | X | Nevada |  | X |  |  |  |  |
| Arkansas |  |  |  |  |  | X | New Hampshire |  | X |  | X |  | X |
| California |  |  |  |  |  |  | New Jersey | X | X | X |  |  |  |
| Colorado |  | X |  |  |  |  | New Mexico |  | X |  | X |  | X |
| Connecticut |  |  |  | X |  |  | New York |  |  |  |  |  |  |
| Delaware | X | X | X | X | X | X | North Carolina |  | X |  | X |  | X |
| District of Columbia | X | X | X | X | X | X | North Dakota |  | X | X | X | X | X |
| Florida |  | X |  |  | X |  | Ohio |  | X |  |  |  |  |
| Georgia |  | X | X |  | X | X | Oklahoma |  |  |  |  |  |  |
| Guam | X | X |  | X |  | X | Oregon |  |  |  |  |  |  |
| Hawaii |  |  |  |  |  |  | Pennsylvania |  | X |  | X |  | X |
| Idaho |  |  |  |  |  |  | Puerto Rico | X |  | X |  | X |  |
| Illinois |  |  |  | X |  |  | Rhode Island |  | X |  |  |  |  |
| Indiana |  | X |  | X | X | X | South Carolina |  | X | X |  |  |  |
| Iowa |  | X | X | X |  | X | South Dakota | X |  | X |  | X |  |
| Kansas |  |  |  |  |  |  | Tennessee |  |  | X |  |  | X |
| Kentucky |  | X |  | X |  | X | Texas |  | X | X | X |  | X |
| Louisiana | X | X |  | X |  |  | Utah |  |  |  |  |  |  |
| Maine |  |  | X | X | X | X | Vermont |  | X |  |  |  |  |
| Maryland |  | X |  | X |  |  | Virgin Islands | X | X |  |  | X | X |
| Massachusetts |  |  |  |  |  |  | Virginia | X | X | X | X | X | X |
| Michigan |  | X |  | X |  | X | Washington |  | X |  |  |  |  |
| Minnesota |  | X |  | X |  | X | West Virginia |  |  |  |  |  |  |
| Mississippi | X |  | X |  | X | X | Wisconsin | X | X | X | X | X | X |
| Missouri |  | X |  | X | X |  | Wyoming | X | X |  | X |  | X |
|  |  |  |  |  |  |  | Total | 13 | 35 | 17 | 26 | 14 | 26 |

1. Potential impact of Covid-19 period on patients with Diabetes

| **Factors** | **Study 1**  **Diabetes-related Self-care Practice Activities**  **Fully Completed** | | | **Study 1**  **Diabetes-related Self-care Practice Activities**  **Partially Completed** | | |
| --- | --- | --- | --- | --- | --- | --- |
|  | **OR** | **95% CI** | **p-value** | **OR** | **95% CI** | **p-value** |
| Interview Year |  |  | 0.71 |  |  | 0.49 |
| *2020* | 0.88 | 0.45, 1.72 |  | 1.01 | 0.53, 1.93 |  |
| *2021* | 1.32 | 0.60, 2.92 |  | 1.40 | 0.66, 2.98 |  |
| *2016 (Ref)* |  |  |  |  |  |  |

| **Factors** | **Study 1**  **Diabetes-related Annual Clinical Care Activities**  **Fully Completed** | | | **Study 1**  **Diabetes-related Annual Clinical Care Activities**  **Partially Completed** | | |
| --- | --- | --- | --- | --- | --- | --- |
|  | **OR** | **95% CI** | **p-value** | **OR** | **95% CI** | **p-value** |
| Interview Year |  |  | 0.70 |  |  | 0.40 |
| *2020* | 0.81 | 0.27, 2.42 |  | 0.91 | 0.31, 2.71 |  |
| *2021* | 0.64 | 0.23, 1.82 |  | 0.68 | 0.24, 1.98 |  |
| *2016 (Ref)* |  |  |  |  |  |  |

1. Potential impact of changes of ADA screening guideline

In sensitivity analyses using the 2021 ADA guideline with an age threshold of 45 years for screening, a significant association was found between race and ethnicity and diabetes screening test uptake (χ^2^ _R-S_=432.2, DF=4.3, p<0.0001).

| **Factors** | **Study 2**  **Diabetes Screening Service**  **Ever Taken a Screening Test**  **within the Past 3 Years** | | |
| --- | --- | --- | --- |
|  | **OR** | **95% CI** | **p-value** |
| Race/Ethnicity |  |  | <.0001 |
| *AA* | 0.56 | 0.53, 0.60 |  |
| *AIAN* | 0.92 | 0.86, 0.98 |  |
| *H/L* | 0.91 | 0.89, 0.94 |  |
| *MRO* | 0.86 | 0.82, 0.90 |  |
| *NHB* | 1.03 | 1.00, 1.06 |  |
| *PI* | 0.71 | 0.64, 0.79 |  |
| *NHW (Ref)* |  |  |  |

**References:**

1. *2021 BRFSS Questionnaire*. Centers for Disease Control and Prevention. June, 2022. Accessed October 10, 2023. https://www.cdc.gov/brfss/questionnaires/pdf-ques/2020-BRFSS-Questionnaire-508.pdf

2. *Calculated Variables in the 2021 Data File of the Behavioral Risk Factor Surveillance System.* Centers for Disease Control and Prevention. June, 2022. Accessed October 10, 2023. https://www.cdc.gov/brfss/annual_data/2021/pdf/2021-calculated-variables-version4-508.pdf
